# Introduction to the special topic: a multidisciplinary approach to motor learning and sensorimotor adaptation

**DOI:** 10.3389/fnhum.2013.00543

**Published:** 2013-09-09

**Authors:** Rachael D. Seidler, Sean K. Meehan

**Affiliations:** ^1^Psychology, Kinesiology, Neuroscience, Neuromotor Behavior Laboratory, University of MichiganAnn Arbor, MI, USA; ^2^Kinesiology, University of MichiganAnn Arbor, MI, USA

**Keywords:** motor learning, sensorimotor adaptation, sequence learning, motor cortex, consolidation

“The plasticity of the living matter of our nervous system, in short, is the reason why we do a thing with difficulty the first time, but soon do it more and more easily, and finally, with sufficient practice, do it semi-mechanically, or with hardly any consciousness at all.” –William James, 1899.

Advances in neuroimaging over the past 20 years have provided insight into the dynamic neural processes underlying human motor skill acquisition, focusing primarily on brain networks that are engaged during early versus late stages of learning. What has been challenging for the field is to tightly link these shifting neural processes with what is known about measureable behavioral changes and strategic processes that occur during learning. The complex nature of behavior and strategy in motor learning often result in a trade-off between experimental control and external validity. Researchers in different disciplines have employed varying approaches to understand motor learning but with relatively little crosstalk. Here, we bring together a set of papers which investigate skill learning spanning multiple domains.

There are several striking and unique features about the papers assembled for this special issue. One is the broad range of investigative techniques brought to bear on the problem of understanding skill acquisition, including cutting edge analytical approaches (Abe and Sternad, 2013; Sami and Miall, 2013), metrics of brain structure and function (Kam et al., 2012; Steele et al., 2012; Bernard and Seidler, 2013; Gentili et al., 2013; Wadden et al., 2013), behavioral experiments with carefully crafted conditions (Armstrong et al., 2013; Kitago et al., 2013; Leow et al., 2013; Nemeth et al., 2013; Taylor and Ivry, 2013), and comprehensive reviews which put forth new theories and novel viewpoints for interpretation (Abrahamse et al., 2013; Bock, 2013; Heuer and Sülzenbrück, 2013; King et al., 2013; Ruddy and Carson, 2013; Vadakkan, 2013). We expect that motor scientists will find inspiring new ideas, techniques, approaches, and theories in this collection of articles.

Another important aspect of these papers is that they report on differing types of skill acquisition including practice of a new skill, adaptation to visuomotor distortions, and acquiring new action sequences. For example, Heuer and Sulzenbruck review their findings evaluating how subjects learn the transformation of a sliding first-order level. This has highly practical implications as this tool type is used in minimal access surgery. The sliding first-order level is a type of tool often used in laproscopic surgery; a fulcrum effect at the skin insertion site results in forward hand movements producing backward tool movements. Moreover, linear hand motions result in curved tool tip paths. Taylor and Ivry leverage comparisons of subjects adapting to visuomotor rotations and to visual translational shifts, and report an interaction between the type of perturbation applied and whether targets are presented in a circular or rectilinear arrangement. Interestingly, they observed that generalization of adaptation across the workspace was linked more to the environmental context than to the perturbation type. Steele and colleagues report findings from a multimodal neuroimaging study using their well-characterized temporal motor sequence task, which requires participants to learn both spatial response locations and a temporal rhythm, similar to playing a musical instrument. They report complementary structural and functional changes with learning; the rate of learning was positively correlated with gray matter volume in cerebellar lobules HV and VI. These same regions exhibit decreases in functional activation with training. Finally, Kitago et al. focus on unlearning in an effort to determine whether it represents forgetting of acquired representations or just reverting back to habitual performance. Their findings support that unlearning is not just forgetting, but is rather an active process. This has important implications for individuals who need to learn new ways of performing everyday skills after suffering from injury or neurological insult.

Several of the papers in this special issue also highlight the differing contributions of neurocognitive mechanisms across learning, consolidation and retention. For example, Nemeth et al. assessed skill learning in healthy adults and those with mild cognitive impairment to investigate the role of the hippocampus and medial temporal lobe (MTL) structures in skilled learning. Using the alternating serial response task (ASRT) they report that individuals with MCI, and likely compromised hippocampal/MTL structures, demonstrate a reduced ability to reactivate/recall learned sequences in subsequent blocks of practice. Interestingly, they report that differences in learning disappeared during the second half of a practice block suggesting a differential role for hippocampus/MTL structures across practice even within a block. In a second paper, Wadden et al. evaluated individual variability in the neural networks underlying motor sequence learning in middle aged adults. Comparing initial task performance to that at a delayed retention test following 5 days of continuous tracking practice they report variability in overall measures of implicit sequence specific learning. However, when learning was decomposed into temporal and spatial elements to account for individual variation, improvement in temporal elements were associated with a network of cortical, sub-cortical and cerebellar areas tied to performance instruction stressing speed over accuracy. In a third paper, Abe and Sternad highlight time dependent changes in learning parameters across six days of a virtual ball throwing task. Analyzing both the distribution and temporal structure of variability they demonstrate and model the importance of time scales. These papers demonstrate that understanding changes across the time course of learning, consolidation and retention is crucial to evaluating the contributions of neurocognitive mechanisms and needs to be investigated despite the difficulty in undertaking such work.

It is our belief that this assemblage of papers will facilitate an integrative view of motor learning, foster discussion across disciplines, and stimulate collaboration. Such a cross disciplinary focus will help to elucidate the neural and cognitive processes underlying skill learning, and may serve to further accelerate translational paradigms that are grounded in skill learning theory.
